# Beliefs, Perceptions, and Behaviors Regarding Chronic Respiratory Diseases of Roma in Crete, Greece: A Qualitative FRESH AIR Study

**DOI:** 10.3389/fpubh.2022.812700

**Published:** 2022-04-15

**Authors:** Marilena Anastasaki, Egid M. van Bree, Evelyn A. Brakema, Ioanna Tsiligianni, Dimitra Sifaki-Pistolla, Vasiliki E. Chatzea, Matty C. Crone, Andreas Karelis, Rianne M. J. J. van der Kleij, Charlotte C. Poot, Ria Reis, Niels H. Chavannes, Christos Lionis

**Affiliations:** ^1^Clinic of Social and Family Medicine, School of Medicine, University of Crete, Heraklion, Greece; ^2^Department of Public Health and Primary Care, Leiden University Medical Centre, Leiden, Netherlands

**Keywords:** Roma, chronic respiratory diseases, health literacy, culture, trust, health behavior, barriers to care, low-resource settings

## Abstract

**Background:**

The global burden of chronic respiratory diseases (CRDs) disproportionally affects Roma populations. Health interventions addressing CRD among Roma or other vulnerable groups often fail to be effective, as their implementation strategy misaligns with the local context. To design context-driven strategies, we studied CRD-related beliefs, perceptions, and behaviors among a Greek Roma population, focussing on asthma and COPD.

**Methods:**

For this qualitative study in Crete, Greece, we used a Rapid Assessment Process. We conducted interviews and focus groups with purposively selected Roma community members (CMs), key informants (KIs) and healthcare professionals (HPs) serving the population. Data were triangulated using observations of households and clinical consultations. Key themes were identified using Thematic Content Analysis. The Health Belief Model, the Explanatory Model of Illness, and the Theory of Planned Behavior that are complementary is some aspects, guided our methodology with the several variables from them to be integrated to better understand CRD risk preventative behavior.

**Results:**

We conducted six focus groups, seven interviews and 13 observations among 15 CMs, four KIs, and three HPs. Five themes emerged: (1) Poor CRD-awareness (smoking and household air pollution were perceived as harmful, but almost exclusively associated with acute rather than chronic symptoms); (2) Low perceived susceptibility to CRD (and CMs tended to ignore respiratory symptoms); (3) High risk exposure (smoking was common, and air pollution was perceived inevitable due to financial constraints); (4) Healthcare seeking (healthcare was sought only for persistent, severe symptoms, daily needs were a priority); (5) Perceived barriers/facilitators to care (health illiteracy, perceived discrimination and financial constraints were main barriers; established trust the main facilitator).

**Conclusion:**

These five themes highlight that strategies to tackle CRD in the studied Roma setting require a multilevel approach: bridging awareness gaps at the population level, providing resources to enhance the adoption of healthy behaviors, and fighting discrimination at the societal level, whilst establishing trusted relationships at the local level. Similar methodologies to address local context may strengthen the implementation of effective interventions for similarly vulnerable and/or low-resource populations.

## Introduction

Chronic respiratory diseases (CRDs) are a major burden to global health (1, 2). According to the World Health Organization, the majority of asthma and more than 90% of chronic obstructive pulmonary disease (COPD)-related deaths occur in low-resource settings (3, 4). The major CRD risk factors are tobacco smoking, household air pollution (HAP), and occupational exposure (2, 5). HAP is caused by biomass fuel burning for cooking or heating, and results in about 3 million deaths annually (6, 7).

Particularly vulnerable to CRDs are Roma (8–10), Europe's largest ethnic minority (11). Especially in older age groups, Roma experience more frequent activity-limiting disabilities and health problems compared to non-Roma (10, 12, 13). These poor health outcomes have been linked to poverty, deprivation, marginalization, and discrimination across multiple European countries (14–16). High rates of tobacco smoking are well-documented and an increased risk of HAP exposure has been suggested (10, 14, 15, 17). Similarly, Roma in Greece were previously found to be at risk of a low health-related quality of life strongly associated with chronic diseases and a reduced lung function due to poor living conditions and exposure to tobacco smoke (16, 18). In Greece, 56.5% of Roma were habitual smokers and 22.0% lived in shanty towns (19). Although recent Roma-specific data in Greece are unavailable, the overall burden and prevalence of CRDs have increased in the wake of austerity measures following the financial crisis (20, 21).

Despite national and European efforts to improve access to care for Roma, health improvements remain limited (22). Previous census among Roma in Greece have indicated a low education level, high uninsured rate, and difficulty in accessing healthcare (23, 24). Aggravated poverty and increased discrimination result from the coronavirus pandemic and measures taken to contain it, disproportionately affecting Roma communities (25, 26). According to the recently published “Roma strategic framework” of the European Commission, 80.0% of Roma are still at risk of poverty and 61.0% experience housing deprivation (27). In terms of general health status, a recent study of over 500 adults living in Roma settlements throughout Greece indicated that, although the majority of participants perceived their health as good/very good, about half had been diagnosed with at least one chronic disease. Socio-economic determinants of health including sex, age and poverty indicators were significantly associated with self-perceived health status and with the presence of a chronic disease (28).

Health interventions developed in affluent settings often fail to be successful when implemented in low-resource settings, such as Roma communities. Evidence is scarce regarding facilitators and obstacles of implementation processes (29). Compatibility with the local context, in particular with health beliefs, attitudes, and behaviors, is crucial for successful implementation of health interventions (30–32). To better understand Roma's vulnerability to CRDs and to develop context-appropriate interventions, insight into their health beliefs, attitudes, and behavior is necessary (15, 33). Therefore, the aim of this study was to explore beliefs, perceptions, and behaviors related to CRDs, and the experienced barriers to healthcare among Roma in Crete, Greece.

## Materials and Methods

### Study Design

This was a qualitative study. The methodological approach was based on the “SETTING” tool for context mapping of health interventions in low-resource settings (34), starting with co-setting study priorities with local stakeholders. Our multidisciplinary team was composed of external and local experts and members of the Roma population. We conducted interviews and focus group discussions (FGDs) with Roma community members (CMs), healthcare professionals (HPs) serving the population and key informants (KIs). Observations of households and clinical consultations were additionally conducted for data triangulation. This study was part of the European Horizon 2020 project “FRESH AIR” (35) (trial registration number NTR5759), targeting the prevention, diagnosis, and treatment of CRDs in low-resource settings. As part of FRESH AIR this study was also conducted in other low-resource populations of Greece, namely in rural settings. This paper follows the COREQ guidelines for reporting qualitative research (36) (Appendix 1).

### Setting

The study took place in the largest Roma camp in Crete with approximately 580 residents. Selection of the study setting was based on convenient preference of a site where the research team or our engaged stakeholders already had established relationships with the communities. Living conditions in the camp are characterized by poor housing and hygiene. Most inhabitants live in improvised constructions (tents and shacks of low-quality materials), while there is no sewage system or electrical network. Although Roma have been tolerated to reside on location for over 10 years, the camp is not recognized as a legal place of residence. According to a census by the local support center (below), half of the adult population has not attended school. Access to the camp by non-Roma is socially challenging, making the population very hard-to-reach. Appendix 2 includes detailed information about the setting and population of this study.

The Support Center for Roma and Minority Groups (SCRMG) is a municipal service that provides basic primary and social care to the Roma community which is subject of this study. Continuous support by medical personnel is lacking, partially due to fluctuating funding. The SCRMG is located in close vicinity to the camp, but regular healthcare services are distant (7–10 km to hospitals and 35 km to the nearest primary care facility).

In Greece, 96.0% of Roma have been reported to live below the country's at-risk-of-poverty threshold, compared with 22.0% of the general population (24). The risk of poverty has not been found to be substantially different across neighborhoods. In Roma youth, 81.0% of women and 38.0% of men report neither work or education as their main activity, compared to 17.0% of Greek youth. Living in dwellings with e.g., damp walls or rot in window frames was documented for 37.0% of Roma compared to 13.7% of the general population (24). Settlements have previously been described to be located on vacant sites with limited to no access of basic amenities and risk of compulsory removal (37). In addition, a strong sense of community and behaviors of introversion and resistance to influences from the outside were mentioned. Hardly any scientific reports exist comparing ways of life and functioning of local support centers between different Roma settlements in Greece. One previous study reported 76% of Roma in settlements to live in permanent houses and found extensiveness of available services between support centers to vary considerably (38).

On a European scale, it is difficult to make a direct comparison of living conditions and socio-cultural values of the study population with other Roma communities given the strong heterogeneity of the European Roma minority (19). However, a general characterization can be made. Recent reporting by the EU Agency for Fundamental Rights has documented 80.0% of Roma to live below their country's at-risk-of-poverty threshold, 10.0% to live in housing without access to electricity, 53.0% of Roma children to participate in compulsory primary education, and only 25% of Roma reporting to be (self)-employed (24). Compared with the general population, more Roma women report “domestic work” as their main activity.

### Participants

Participants were selected by a combination of purposive and convenience sampling. As the study aim was explorative, we aimed for a diverse sample in terms of sex, age and background. We included:

▪ CMs: Any Adult Camp Resident (≥18 years old).
▪ HPs: Any health professional working with Roma CMs in the camp.
▪ KIs: Any relevant stakeholder with either in-depth knowledge or an overview of beliefs, perceptions and behaviors of the camp population.

Apart from the age criterion (≥18 years old), no other inclusion criteria were employed for sample selection. People living outside the camp and not in direct contact with the population, or people unable to participate due to physical or mental disabilities, were excluded.

For participant inclusion, we first engaged with the SCRMG. Over the years, these professionals have built trust with the Roma population. SCRMG professionals were firstly included as HPs or KIs in the study. Afterwards, they accompanied us inside the camp to facilitate trust from the CMs. A mediator, who was a member of the Roma community and SCRMG collaborator, ensured smooth and effective access to residences. Sample size was intended to be guided by data saturation, yet in execution dependent on CM's willingness to participate and time availability of SCRMG-professionals (see discussion).

### Theoretical Framework

**Figure 1:**
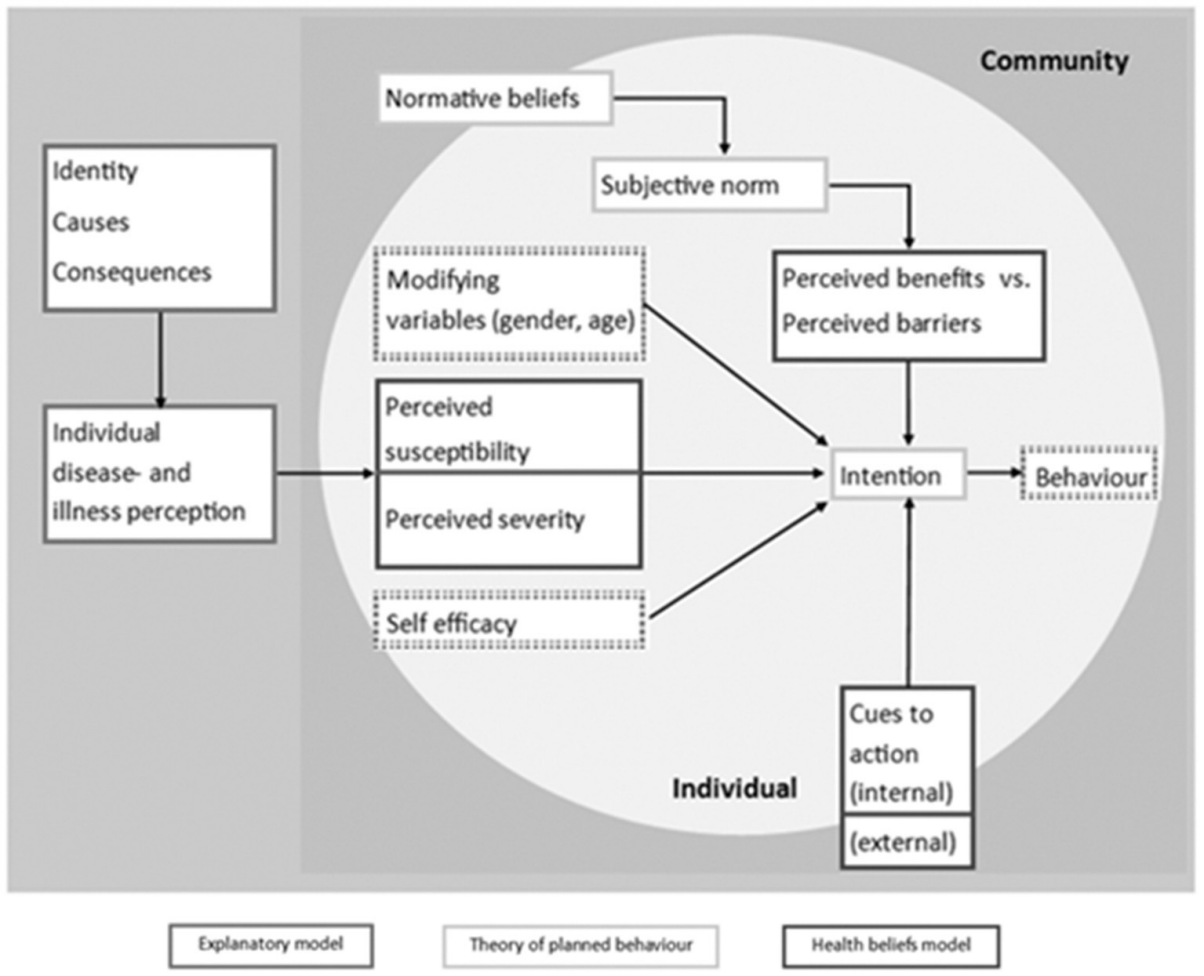
The combined theoretical framework (reprinted with permission), as applied in the SETTINGtool (33).

Our theoretical framework was based on a combination of three health behavior models: the Health Belief Model, the Explanatory Model of Illness, and the Theory of Planned Behavior (39–41). The framework consisted of elements including perceptions of CRD identity, susceptibility, barriers toward behavioral change and risk reduction, help seeking behavior by CMs, and helping behavior by HPs. All study materials (topic guides, observation forms, surveys) were guided by this framework (Figure 1, Appendix 3 detailing the reasoning behind development and use).

### Data Collection

We simultaneously conducted semi-structured interviews, FGDs and observations in September 2016, using the Rapid Assessment Process (RAP) (42). Our initial intention was to perform a mixed-methods study including questionnaires to quantify CRD risk factor exposure and frequency of perceived potential causes, yet in execution found willingness to participate, time availability and practical aspects to strongly influence our ability to collect responses. Therefore, during the RAP, we decided to use the obtained questionnaires for data triangulation rather than analyse them separately. Within the RAP, researchers immersed into the community for 2 days in total and collected data in a concise and time-efficient way. CM interviews, FGDs, and household observations took place inside the camp. Interviews with HPs and KIs and observations of clinical consultations were conducted in SCRMG facilities. Two Greek and one Dutch interviewer conducted each activity. No CMs other than participants were present during interviews or FGDs.

Study purposes and interviewers' backgrounds were explained to CMs by SCRMG professionals. During research activities, interviewers also introduced themselves, explained research objectives, and addressed confidentiality. Participants were provided with a study information sheet, while explicit information and opportunity to ask questions were provided verbally. All individuals provided signed informed consent before participation. The study had been approved by the 7th Health Region of Crete (6951;27/05/2016) and the Leiden University Medical Center Medical Ethical Committee (P16.063;04/15/2016) prior to its initiation.

Following the RAP (42), data were preliminarily analyzed at several time points. This allowed for iterative adjustment of the data collection strategy, ensuring that it was driven by local developments and research needs.

Based on the theoretical model, topic guides were developed for data collection (Appendix 4). Since we aimed at exploring participants' own perceptions, no particular definition for chronic respiratory disease was provided to them during interviews. However, our approach was mainly focused on asthma and COPD, which were introduced to participants through a vignette describing a typical case of an individual with chronic respiratory symptoms (Appendix 4). Starting with the presentation of this vignette, topic guides prompted participants' reflections on presented respiratory symptoms, perceived causes and implications, beliefs and behaviors toward risk reduction, helping behaviors, and healthcare-seeking. Activities were audio-recorded if participants gave consent to do so, and field notes were taken. Interviews and FGDs lasted approximately 1 h. Debriefings with the research team occurred after each activity to allow further adjustments.

### Research Team

The research team comprised of two Dutch and five Greek researchers, both male and female. The participation of SCRMG professionals and one mediator from the Roma community helped facilitate trust of the Roma toward researchers. Interviews and FGDs were conducted in Greek, with Greek team members interpreting for Dutch researchers. Before starting the RAP, all researchers were extensively trained by the leading expert researcher (E.B.).

Interviewers had no previous relationship with participants. The SCRMG employees were professionally related to CMs.

### Data Analysis

Audio-recordings were transcribed verbatim and translated to English before coding. An inductive-deductive approach was used for transcript analysis. Specifically, transcript coding was both open and deductive, following the combined theoretical framework applied in this study, while allowing for new emergent themes and concepts. Coding and analysis were conducted manually and individually by two researchers (E.v.B. and M.A.) using Microsoft Word version 16. Discrepancies were solved through discussion until consensus was reached. Data structuring and reduction was first performed per informant group (CMs, HPs, and KIs), before combining the data of the different groups. Subsequently, emerging themes were identified using Thematic Content Analysis by E.v.B. and M.A., supervised by E.B. (43). Findings from observations, and field notes were then studied for presence of additional or contradicting themes to triangulate data before final themes were concluded.

## Results

Twenty-two participants (15 CMs, 3 HPs, 4 KIs) were included. In particular, interviewed HPs were doctors and nurses, while KIs were population mediators and social care professionals. We also collected three CM questionnaires which were used for data triangulation. They contained information on risk exposure and perceived causes of CRDs which did not contrast or hold any new information compared to the information obtained during the interviews and FGDs

As shown in Table 1, female sex prevailed our sample (14/15 CMs, 2/3 HPs and 3/4 KIs). The age range was 20–60 years for CMs, 30–50 years for HPs and 30–50 years for KIs. Seven interviews and six FGDs were conducted. Additionally,13 observations were performed. For one interview and two FGDs CMs did not consent to audio-recording. In these cases, the findings were documented in field notes and included in preliminary analysis during researcher debriefings.

**Table 1:** Basic sample characteristics and field activities performed.

	**Community** **members**	**Healthcare** **professionals**	**Key** **informants**
**Number of individuals**	15	3	4
*Male*	1	1	1
*Female*	14	2	3
*Age range*	20–60	30–50	30–50
Focus groups	5	–	1
Interviews	2	3	2
Observations*	7	6	-

* *Household observations for community members and consultation observations for healthcare professionals*.

*All observed consultations were performed by a single healthcare professional*.

During our fieldwork, the aforementioned poor living conditions were confirmed. Lack of electricity was reported as a major problem by all encountered camp residents and as a frequent reason of conflict with local authorities (Appendix 2). As elaborated on in the Results section below and Appendix 2, lack of electricity was also linked to health behavior as it prevented, for example, the proper practice of hygiene and proper medication storage. In terms of exposure to risk factors, tobacco smoking was observed and reported at high frequencies during our camp visits. Additionally, indications related to increased levels of household air pollution were documented, as improvised biomass-burning heating devices were observed during almost all our household visits, while burning materials (wood, melamine pieces, plastics and others) were seen stored outside of many households throughout the camp.

Themes emerging from the analysis are presented in Table 2 and elaborated on below, based on reflections of the combining elements of our theoretical framework. Appendix 5 presents additional quotes supporting findings or providing contextual information.

**Table 2:** Overview of key themes.

**1. CRD-related awareness and beliefs**
- Limited awareness of CRDs and long-term exposure to risk factors
- CM's beliefs and perceptions connected to acute symptoms (e.g. coughing, runny nose)
**2. Perceived severity and susceptibility**
- Respiratory health is perceived as important, peers are urged to seek care
- Low perceived disease susceptibility, especially among young Roma
**3. Risk exposure**
- High prevalence of smoking and limited intention to quit, despite known harmful effects
- HAP caused by widespread woodstove usage, attributed to financial constraints and poor living conditions
**4. Healthcare seeking behavior**
- Daily needs and the perception of being strong are prioritized over personal health, especially among men
- Persistent and activity-hampering symptoms drive help seeking behavior
**5. Perceived barriers/facilitators to care**
- Health illiteracy, experienced discrimination, and financial constrains as barriers to care
- Established trust and continuity of support services as facilitators to care

### CRD-Related Awareness and Beliefs

In terms of perceived disease identity, beliefs expressed by CMs were predominantly connected to acute respiratory symptoms, such as coughing and a runny nose, rather than chronic symptoms. While medical respiratory terminology (such as dyspnoea) was hardly used, asthma was a familiar term. However, the meanings attached to asthma (e.g., the chronic nature of the disease and its health implications) differed from medical connotations.

▪ *Interviewer: Asthma... have you heard about it?*
▪ *All CMs: Yes..*.
▪ *CM9: This disease where you use a mask..*.
▪ *CM8: It's mostly for older people... (CMs, female, age 20–30, in FGD)*

In contrast, COPD was completely unknown (Appendix 4).

In terms of perceived causes, CMs believed several factors to influence respiratory health. Heredity, aging, allergies, and exposure to observable risk factors were mentioned as potential causes, such as tobacco smoke and HAP from burning low-quality fuels (Appendix 3). Triangulation data showed similar findings.

“*(…) when we light the fireplace, and smoke is coming out... Maybe the cause [of breathlessness and productive coughing] is having the fire inside... (CM1, female, age 31)”*

“*I believe that it comes with age... Because everything appears when you get old... And from smoking! There are some people that when they have it [i.e. the lung problem described in the vignette] they quit smoking. (CM8, female)”*

However, according to a HP, comprehensive understanding of CMs regarding the medical aspects of CRD and the long-term effects of exposure to risk factors was limited:

“*A kid may catch a cold more frequently than other children, because his or her respiratory system is no longer in a good condition due to smoke from the woodstove (…) even if you explain this [to Roma], they can't conceive it. (HP3, female)”*

According to CMs, the prevalence of CRD in the camp was low. HPs confirmed this, yet cautioned that it could be underdiagnosed. Circulatory, metabolic and mental health disorders were noted among the most frequently encountered conditions in the camp:

“*We may see problems of diabetes, blood pressure, heart problems. Respiratory problems not so much. (KI1, female)”*

“*(…) the percentage of people that suffer from respiratory diseases is not high… there is the possibility of not knowing the exact number [of respiratory patients] because they do not make appointments or undergo medical examinations, especially regarding respiratory issues. (HP2, female).”*

“*The first thing, that comes to mind, as main [..] basic issue they seek to solve, is their psychiatric problems. (KI3, female)”*

### Perceived Severity and Susceptibility

In agreement with the components of our theoretical framework, individual disease and illness perception of the studied population were linked to perceived susceptibility and perceived severity, which were further linked to action cues and, ultimately, behaviors. According to CMs, Roma perceived respiratory health as important. If a CM experienced persisting symptoms, peers would usually consider this important enough to advise them to visit a doctor, reflecting a subjective norm toward healthcare seeking behavior.

“*(…) when I see someone who coughs and doesn't stop, I tell him to visit a doctor, without waiting, because something may happen to his lungs and he (may) suffer more. (CM3, female, age 45)”*

“*Because everything comes from the lung... All people should take care... maybe your breath will stop... this is what I know... (CM8, female)”*

For personal health issues, however, lower perceived disease susceptibility was mentioned. According to CMs and HPs, especially young Roma tended to ignore respiratory symptoms and rather waited for them to pass, suggesting that age can be a modifying variable toward this direction.

“*As far as their own health is concerned, younger ones are more relaxed... They believe they will never get something. (HP3, female)”*

Symptoms among children were perceived as more alarming. Children's health was clearly prioritized over adults' health (Appendix 5).

“*I don't leave my child without going to the doctor (…) whatever the doctor says, I do everything (…) If I have 100 euros, I will prefer to give them for my kid (...) For myself, I will go to the hospital. But (…) even if I don't go, I have no problem. (CM15, female)”*

### Risk Exposure

Tobacco smoking and exposure to HAP were identified as core risk behavior variables. Namely, in terms of perceptions related to these risk behaviors, all participants (smokers or non-smokers) described smoking to be harmful for health, particularly for smokers (active smoking) and children (passive smoking). Participants consistently reported Roma to be heavy smokers, starting at an early age. Sex seemed to be an important modifying variable for smoking behavior. In particular, although our sample consisted mainly of women, both male and female participants reported that smoking was much more frequent and heavier among men than among women. Passive smoking was recognized to happen frequently. Both CMs and HPs mentioned that the intention to stop smoking was extremely low. Smoking appeared to hold strong emotional value.

“*(…) the doctors refer them [men who smoke] to cessation clinics and we book their appointments, yet they never go. (…) They declare: “I don't want to quit! Do whatever you want, I will not quit!”. (...) They think it's something that makes them forget their troubles, as they say. (HP3, female)”*

“*Interviewer: Do most people smoke inside or outside the house?”*

“*CM14 (female): Inside. Our babies also smoke inside [i.e. refers to passive smoking].”*

HAP, however, appeared to be strongly related to financial constraints, poor living conditions and low health literacy, indicating low socioeconomic status as a significant modifying variable of health behavior. CMs explained that they usually burned cheap, low quality wooden pallets indoors for heating. According to HPs and KIs, CMs' awareness regarding the long-term consequences of HAP for respiratory health may be generally low. HPs and CMs described that “clean” alternatives were too expensive for most CMs and that electricity was unavailable. Especially in winter, ventilation was limited to a minimum to keep the poorly insulated houses warm, leading to high HAP exposure. The presence of indoor woodstoves and wooden pallets throughout the camp was confirmed in our household observations.

“*There are important fears for health during winter with the woodstoves. We [CMs] are naive to be using melamine woods [synthetic woods] (...) We don't have to pay for them, and unfortunately, we can't conceive there will be a problem. (KI2, male, about CMs)”*

“*Interviewer: Do you try to ventilate the house? (…)”*

“*CM3 (female): We don't take much care of it. I close the windows and the door sometimes (…) We all sit close to the woodstove. And I don't open something to ventilate the area. Only when the weather is good….”*

### Healthcare Seeking Behavior

Internal and external cues to action were reflected in the healthcare seeking behavior of the studied population. In particular, all participants mentioned that CMs usually prioritized their daily needs over personal long-term health. Additionally, a generalized negative attitude toward visiting doctors and healthcare services was expressed, attributed mainly to fear of the unknown consequences of a potential diagnosis. As such, healthcare seeking was driven by perceived disease severity, wherein only persistent and activity-hampering symptoms were a direct cue to action.

“*If he has something that needs rest he will not go [to work], but if it is not something serious he will go. Most people go [to work] in order to feed their families. (CM8, female, age 38)”*

“*(…) health is not important for them [i.e. CMs]. It is not a priority. What's important for them is to work, make some money, and have food today. (HP2, female)”*

Interviewed KIs and CMs and fieldnotes, including one male perspective, also suggested that men tend to be more reluctant to talk about disease and visit healthcare services than women. Interviewed participants (both male and female) attributed this directly to men's ability to work and support their families.

“*Because at home, he [the man] is the pillar. And if he had something, he wouldn't be able to work. And if he would see that his children should work [to provide for the family], he would get more worried. (CM3, female, age 45)”*

“*(A Roma man may be like) “If I go to the hospital, they may find that I have a severe condition. I will not go, I will stay at home whatever happens” (...) Or they may fear the hospital and whenever they go they may feel bad. (CM15, female)”*

In general, (male) sex was a significant modifying variable for healthcare seeking practice, since all participants described an important role for men in the Roma community. Due to the majority of interviewed participants being women, this information was largely a secondary report. However, both CMs and KIs mentioned masculine elements of strength and pride to influence help-seeking behavior (norms).

“*In younger ages, the man feels more vulnerable when more people learn that he has a problem (...) Especially if he has not had children yet, this gives him a bad reputation (...) If he is of older age and has had children, it's a matter of the position he has in”*

“*the community (...) he will try to deal with it like “Ok, I have nothing”, but he will always have the fear of losing his prestige. (KI1, female)”*

### Perceived Barriers/Facilitators to Care

As illustrated in our theoretical framework, perceived barriers and facilitators were linked to health care seeking behavior in the studied Roma population. Namely, HPs and KIs perceived (health) illiteracy to be an important barrier to healthcare, limiting the population to understand information and to follow medical advice. Similarly, CMs indicated difficulty to seek medical care.

“*This [illiteracy] (…) is important… it's often a reason for them to have fear, anxiety of cooperating [with HPs], low compliance, or not understanding instructions. For example (…) the importance of participating in a health education [activity] for respiratory diseases. (KI3, female)”*

“*(…) they feel uncomfortable and disadvantaged because they are illiterate, to visit a [health] service and undergo some tasks. Their level of education, I think, draws them back because they feel uncomfortable and disadvantaged. (HP2, female)”*

Also, CMs reported that they experience discrimination in hospitals and receive limited explanations by doctors as significant factors contributing to their reluctance to seek care.

“*(…) some people are racists! (…) Even when I go to hospitals, and I wait in line and it's my turn to go in, they will not call me, they will call their people, their acquaintances (…) That's why we got sick of hospitals... (CM14, female, age 55)”*

Community support during sickness was reported to be strong, such as CMs accompanying peers to healthcare services (subjective norm). The main facilitator to access care was the trust established over the years between the Roma population and the SCRMG facility, increasing HPs' and KIs' influence in supporting and motivating CMs in their health behavior.

“*It's important that they [CMs] trust you. If they don't trust you, the problem will be the same… It is important for these people to trust this [entire] system around them… to have better contact and to accept the message. (HP1, male)”*

Limited financial means and poor living conditions were mentioned as key barriers preventing adoption of healthy practices, wherein financial constraints also limit the ability to overcome the physical distance to healthcare facilities.

## Discussion

### Main Findings

This qualitative exploration of beliefs, perceptions, and behaviors among a Greek Roma population demonstrates five key themes related to CRDs. Concepts around asthma and COPD were introduced to interviewed participants through a vignette. The first emerging theme was related to the components of perceived disease identity and causes and reflected the limited awareness of CRDs and the harm of long-term exposure to risk factors. CM's beliefs and perceptions were predominantly related to acute symptoms. The second theme reflected the perceived severity and susceptibility to CRDs. Although respiratory health was perceived as important, it was reported that especially young Roma tend to believe that their personal susceptibility to CRD is low. The third theme was related to risk behaviors and related perceptions. Tobacco smoking was highly prevalent and intention to quit was low, although it was recognized as harmful for health. HAP due to widespread woodstove usage was attributed to financial constraints and poor living conditions. The fourth theme was related to healthcare seeking behavior and suggested the prioritization of daily needs over health issues. Providing for one's family and the perception of being strong were prioritized over personal health, particularly in men, unless symptoms were persistent and severe (norms). The fifth and last theme indicated the factors influencing access to care and healthcare seeking behavior. Health illiteracy, experienced discrimination, and financial constraints were the main barriers to care and behavioral change. Established trust and continuity of support services were key facilitators to healthcare seeking behavior.

### Interpretation of Findings in Relation to Literature

Respiratory health was considered important by the Roma population of our study, yet did not result in an actual priority due to a lack of awareness about CRD and its risk factors, and a lack of means to reduce exposure. This is a finding that has not previously been reported. We also observed a low perceived disease susceptibility which, according to literature, likely contributes to late presentation at healthcare services and low engagement to preventive activities, especially among male Roma (44, 45). The particular concern about children's health found in this research has earlier been described similarly concerning immunization uptake (46). In line with other studies in different Roma populations, we identified an interplay of day-to-day priorities, ignorance of long-term implications, fear of a diagnosis, distance to healthcare facilities, low levels of health literacy, discrimination-fueled distrust of healthcare providers, and a strong masculine culture to negatively influence health behavior (45, 47–49). Our study is the first, however, to relate this interplay specifically to CRDs and potentially other chronic diseases.

In contrast to previous studies that report a socially bonding “norm” to reject healthcare standards (47, 50, 51), we found that this Roma population urged each other to seek medical care for persisting respiratory symptoms. This may indicate a certain trust developed over the years in local health services, and particularly in the SCRMG (44). Strikingly, this Roma population had more negative attitudes toward hospital care compared to the SCRMG. We noted that previous experiences shaped help-seeking behavior, wherein perceived discrimination negatively influenced behavior. It has been suggested that Roma “nonadherence norms” to medical recommendations are less likely to be present in environments that hold less anti-Roma views (52). This underlines the importance to support non-Roma HPs in their interaction with Roma, as expressed in previous studies (47–49, 51). Considering that most CRD-related care in Greece takes place in hospital settings (52), the importance of welcoming interactions should be duly noted. Identified barriers and facilitators to care, including perceived discrimination, low levels of health literacy, and established trust between the population and the SCRMG, corroborate previous evidence in other Roma populations (44–46, 53).

Furthermore, respiratory risk factors including smoking and HAP were highly prevalent in the Roma population of our study. We uncovered a close relationship between woodstove usage, financial constraints, limited ventilation, and poor living conditions. The negative health effects of the resulting HAP have been broadly reported in literature as a result of substandard infrastructure and poverty among Roma (16, 46, 53). In line with our findings, reluctance toward smoking cessation has been associated with emotional value, low health literacy, and a lack of confidence in effectiveness of risk reduction (54–56). Corresponding widespread smoking, especially in Roma men, is well-documented (15, 18, 19). Other studies have found smoking among Roma to be initiated at a young age and, alike HAP in our study, indicate a strong association with a lower socio-economic status (57, 58).

### Strengths and Limitations

To the best of our knowledge, the perception of respiratory health and associated symptoms, such as coughing, by Roma has not been studied before. The main strength of our research is its multi-faceted exploration of an undocumented topic based on a well-theorized framework. To achieve an adequate representation of the topic, we collected data from three different stakeholder groups, while combining interviews and FGDs with observations. Transparency and validity in data analyses were promoted through careful thematic analysis by two independent researchers from different backgrounds.

Several limitations merit emphasis, however. First, since this was a hard-to-reach population, we were bound to the limited time available by the SCRMG-professionals to gain CMs' trust. Therefore, we did not achieve the desired sample diversity in terms of sex nor reach full data saturation or collect a sufficient number of questionnaires; the topics low perceived disease susceptibility, experienced discrimination in hospitals, and specifically Roma male views on health and health intervention participation would benefit from further exploration. Nevertheless, based on our current findings, we did not find indications for specific differences between males and females. Second, the sample's dependence on participants' opportunity and willingness possibly created selection bias toward more collaborative Roma. To a certain degree, triangulating interview and FGD data with observations and field notes accounted for this effect. In addition, the multidisciplinary team and inclusion of views from multiple informant groups helped to limit this potential bias. Third, given the reported diversity of Roma as ethnic group, data of this study reflecting one camp, and the qualitative study design, findings cannot be generalized. Rather, they should be interpreted with caution and in relation to previously published work (19, 37). Finally, given that the Roma population is culturally distinguished from the rest of FRESH AIRs' low-resource implementation settings, our theoretical framework would have been benefited by a sociology of health perspective, especially with regard to social contexts of poverty or strong ethnic differentiation.

### Implications for Research, Policy, and Practice

Our study demonstrated typical beliefs, perceptions, and behaviors among a Greek Roma population, which should be considered during provision of care to ensure equity in access to and quality of care. Our findings suggest that successful approaches to combat CRD among Roma would benefit from following a multidisciplinary approach starting from the population and expanding toward the societal and public health level, as elaborated below. Similar approaches could be considered for other chronic diseases and vulnerable or low-resource populations. Such strategies would be both relevant and timely, as structural primary care reforms have been unfolding in Greece and the urgency of Roma inclusion has been underlined by the recent EU Roma strategic framework (27). Above all, our findings emphasize the importance to address these local beliefs and behaviors to combat the poor health outcomes of Europe's largest ethnic minority, which was also disproportionately hit by the coronavirus pandemic (8, 15, 16, 25, 26, 45).

At the population level, there seems to be a general need to improve CRD-related health literacy among Roma. Improved awareness of the harmful effects of HAP and other risk factors should be developed to aid conceptualization of CRDs and to weigh long-term implications and daily priorities. This is particularly important, considering that COPD poses a significant burden in Greece (20, 21, 59, 60) and taking into account Roma's vulnerability to CRDs (8–10). Although improvement of health literacy in itself is an important focus, policy makers should go beyond and actively work with cultural differences related to health and local reality (53, 56, 61, 62). This can only happen in collaboration with Roma: leveraging trust established by local mediators and strengthening relationships between Roma communities and primary care services (49, 63). For example, a Hungarian primary care programme involved non-professional health mediators of Roma ethnicity to promote access to care and was recently positively evaluated (64). CRD-specific examples of population-level interventions to increase CRD-related health literacy through increasing awareness can be drawn from FRESH AIR's work in global low-resource settings. Such approaches have used implementation science to culturally adapt and implement evidence-based cascading train-the-trainer awareness-raising interventions regarding the damaging effects of smoke from tobacco and HAP. These interventions start by identifying context-specific factors that may drive effectiveness and engage the whole community and relevant stakeholders to their development and delivery. Their effects on knowledge increase have been proven promising (65, 66).

In addition, a strong masculine culture was reflected in this study by all interviewed participants (regardless of their sex) and this may be a point of attention. Although further studies would be beneficial to better understand male perspectives on health behavior, previous studies have also indicated a strong culture of masculinity to hamper smoking cessation and participation in cancer screening among Roma elsewhere in Europe (56, 67). It has also been suggested that Roma are much less likely to support tobacco control measures than non-Roma of similar socioeconomic status (54, 67). Although evidence is limited, implementation strategies of risk-reduction progammes, such as smoking cessation, may benefit from taking into account such different attitudes and employing culturally acceptable methods to address the population.

Expanding toward the societal and public health level, Roma's living conditions and access to care need to be addressed. Structural improvement of (financial) access to better housing and cleaner fuels is necessary to enable behavioral change and reduce HAP. Within the studied population, socio-economic status was an important modifying variable for health seeking behavior. As appears from reporting by the EU Agency for Fundamental Rights, Roma, such as the population residing in the camp subject of this study, face precarious work, limited state support in provision of needs such as electricity, and uncertainty of long-term residence (25). Own investments in improvement of living conditions can therefore be perceived as too expensive, especially in the light of an uncertain duration of stay.

Furthermore, community support centers can be short of (medical) personnel and subject to fluctuating funding (69). Although this theme was not discussed in the Results section of this manuscript, additional quotes and context information (Appendix 5) do refer to the subject of fluctuating financial means. Only using available funds to facilitate direct care provision may be insufficient. Public health outreaches have been found to reinforce disengagement and do not alter the underlying system shaping health behavior (45, 53). Therefore, to facilitate adequate help-seeking behavior, healthcare efforts could benefit from taking a systemic approach, including measures to improve mutual cultural awareness between Roma and non-Roma (62, 70). A community-based participatory approach has been suggested as an effective way to foster Roma-involvement in readjustment of community agendas and allocation of resources based on local priorities (51, 69). As time investment can be substantial and expensive, university involvement or public-private partnerships have been suggested as successful enablers (68, 70).

## Conclusion

Respiratory health does not seem an actual priority for the studied Roma population, despite its reported perceived importance. Health illiteracy and awareness of long-term effects of CRDs and their risk factors, such as HAP, need to be enhanced. To enable adequate help-seeking behavior and behavioral change benefiting health, implementation strategies of CRD-related interventions could increase their success by including investments in establishing trust, fostering community engagement, identifying ways to minimize the effects of financial constraints, and ensuring welcoming interactions between Roma and non-Roma. Strong relationships between Roma communities and primary care providers, such as the municipal support center and Roma mediator in this study, can act as an important facilitator. These findings can be used to design more successful context-driven implementation strategies for evidence-based respiratory health interventions for the studied Roma population and potentially for other Greek or European Roma populations. This study's approach and our findings may be relevant, not only for CRD and Roma, but also for other chronic diseases and similarly vulnerable/low-resource populations.

## Data Availability Statement

The raw data supporting the conclusions of this article will be made available by the authors, without undue reservation.

## Ethics Statement

The studies involving human participants were reviewed and approved by the 7th Health Region of Crete (6951; 27/05/2016) and the Leiden University Medical Center Medical Ethical Committee (P16.063; 04/15/2016). The patients/participants provided their written informed consent to participate in this study.

## Fresh Air Collaborators

Pham Le An, Azamat Akylbekov, Andy Barton, Antonios Bertsias, Pham Duong Uyen Binh, Job FM van Boven, Dennis Burges, Lucy Cartwright, Liza Cragg, Tran Ngoc Dang, Ilyas Dautov, Berik Emilov, Irene Ferarrio, Frederik A van Gemert, Ben Hedrick, Le Huynh Thi Cam Hong, Nick Hopkinson, Elvira Isaeva, Rupert Jones, Corina de Jong, Sanne van Kampen, Winceslaus Katagira, Bruce Kirenga, Jesper Kjærgaard, Rianne MJJ van der Kleij, Janwillem Kocks, Le Thi Tuyet Lan, Tran Thanh Duv Linh, Kim Xuan Loan, Maamed Mademilov, Andy McEwen, Patrick Musinguzi, Rebecca Nantanda, Grace Ndeezi, Sophia Papadakis, Hilary Pinnock, Jillian Pooler, Maarten J Postma, Anja Poulsen, Pippa Powell, Nguyen Nhat Quynh, Susanne Reventlow, Sally Singh, Talant Sooronbaev, Jaime Correia de Sousa, James Stout, Marianne Stubbe Østergaard, Aizhamal Tabyshova, Tran Diep Tuan, James Tumwine, Le Thanh Van, Nguyen Nhu Vinh, Simon Walusimbi, Louise Warren, Sian Williams.

## Author Contributions

MA participated in local study development and contributed to data collection and analysis. MA and EBre analysed the data, co-wrote the first and subsequent versions of the manuscript. EBra designed the study, coordinated and conducted data collection, gave input throughout the entire data analysis, and writing process. IT participated in the writing of the original FRESH AIR proposal, provided scientific input for local study development, implementation, and results interpretation DS-P, VC, AK, and CP participated in local study development, data collection, and data interpretation. CP, RK, MC, NC, and RR advised throughout the study design and data collection. CL was the country lead for the FRESH AIR project, providing overall supervision and scientific input for study design, implementation, data interpretation, and writing of the manuscript. NC was the principal investigator of the overall FRESH AIR project, an original author of the FRESH AIR proposal, and contributed to the development of the study protocol. All authors critically reviewed the manuscript and approved its final version.

## Funding

This study was funded by the EU Research and Innovation program Horizon2020 (Health, Medical research and the challenge of aging) under grant agreement number 680997 and trial registration number NTR5759. The funders had no role in study design, data collection, data analysis, data interpretation, or writing of the report.

## Conflict of Interest

The authors declare that the research was conducted in the absence of any commercial or financial relationships that could be construed as a potential conflict of interest.

## Publisher's Note

All claims expressed in this article are solely those of the authors and do not necessarily represent those of their affiliated organizations, or those of the publisher, the editors and the reviewers. Any product that may be evaluated in this article, or claim that may be made by its manufacturer, is not guaranteed or endorsed by the publisher.
