# Supplementary material for: Beliefs, Perceptions, and Behaviors Regarding Chronic Respiratory Diseases of Roma in Crete, Greece: A Qualitative FRESH AIR Study
Source: Front Public Health. 2022 Apr 15;10:812700. doi: 10.3389/fpubh.2022.812700 (PMC9051233; doi:10.3389/fpubh.2022.812700)
Supplement: Supplementary file 1 [file Data_Sheet_1.PDF]

Supplementary Material

Beliefs, perceptions, and behaviors regarding chronic respiratory diseases of Roma in Crete, Greece: a qualitative FRESH AIR study

Appendix 1. Completed COREQ-checklist [35] for reporting of this study.

Topic	Item No.	Guide Questions/Description	Reported on Page No.
**Domain 1: Research team and reflexivity**			
Personal characteristics			
Interviewer/facilitator	1	Which author/s conducted the interview or focus group?	6, 10
Credentials	2	What were the researcher's credentials? E.g. PhD, MD	N/A
Occupation	3	What was their occupation at the time of the study?	N/A
Gender	4	Was the researcher male or female?	4
Experience and training	5	What experience or training did the researcher have?	4
Relationship with participants			
Relationship established	6	Was a relationship established prior to study commencement?	3-4, 10-11
Participant knowledge of the interviewer	7	What did the participants know about the researcher? e.g. personal goals, reasons for doing the research	10-11
Interviewer characteristics	8	What characteristics were reported about the interviewer/facilitator? e.g. Bias, assumptions, reasons and interests in the research topic	4, 8, 11
**Domain 2: Study design**			
Theoretical framework			
Methodological orientation and Theory	9	What methodological orientation was stated to underpin the study? e.g. grounded theory, discourse analysis, ethnography, phenomenology, content analysis	3
Participant selection			
Sampling	10	How were participants selected? e.g. purposive, convenience, consecutive, snowball	3, 8-9
Method of approach	11	How were participants approached? e.g. face-to-face, telephone, mail, email	3-4
Sample size	12	How many participants were in the study?	4-5
Non-participation	13	How many people refused to participate or dropped out? Reasons?	4
Setting			
Setting of data collection	14	Where was the data collected? e.g. home, clinic, workplace	2-3

Presence of non-participants	15	Was anyone else present besides the participants and researchers?	4
Description of sample	16	What are the important characteristics of the sample? e.g. demographic data, date	2-3, 4-5, appendix 2
Data collection			
Interview guide	17	Were questions, prompts, guides provided by the authors? Was it pilot tested?	2, appendix 6
Repeat interviews	18	Were repeat inter views carried out? If yes, how many?	N/A
Audio/visual recording	19	Did the research use audio or visual recording to collect the data?	4-5
Field notes	20	Were field notes made during and/or after the interview or focus group?	4
Duration	21	What was the duration of the inter views or focus group?	4
Data saturation	22	Was data saturation discussed?	3, 9
Transcripts returned	23	Were transcripts returned to participants for comment and/or	N/A

Topic	Item No.	Guide Questions/Description	Reported on Page No.
		correction?	
**Domain 3: analysis and findings**			
Data analysis			
Number of data coders	24	How many data coders coded the data?	4
Description of the coding tree	25	Did authors provide a description of the coding tree?	4
Derivation of themes	26	Were themes identified in advance or derived from the data?	4
Software	27	What software, if applicable, was used to manage the data?	4
Participant checking	28	Did participants provide feedback on the findings?	N/A
Reporting			
Quotations presented	29	Were participant quotations presented to illustrate the themes/findings? Was each quotation identified? e.g. participant number	5-7, appendix 6
Data and findings consistent	30	Was there consistency between the data presented and the findings?	5-7, appendix 6
Clarity of major themes	31	Were major themes clearly presented in the findings?	5-7, appendix 6
Clarity of minor themes	32	Is there a description of diverse cases or discussion of minor themes?	5-7, 8, appendix 2 & 6

Appendix 2. Context information on the Roma population, support centre, and camp based on information available prior to research and added information after execution of the study.

A. Context information known prior to research

Data for this study were collected in the largest Roma camp in the region of Crete. Based on the May 2014 annual registration, 528 Roma reside in the camp and 56 in the neighbourhoods of the city. The group consists of 301 adults (159 female, 142 male) and 283 children. The age distribution is: 0-14 years (40.8%), 15-24 years (17.6%), 25-54 years (31.5%) and >55 years (10.1%). Greek is the main language for communication, with 65% only using Greek and 35% using both Greek and Romani. Inhabitants are exclusively Greek Roma and the majority reside in the camp for >20 years. Employment rate is 22%, with an additional 10% of Roma assisting a person who is employed. The declared income of 90% of Roma is below poverty line (up to €6,000 per year), however, 80% of Roma are estimated to end up in a higher range (€9,000-€13,000 per year) when including hidden income. An estimated 50% of Roma have not attended school. During the time of data collection, the Roma population was facilitated by the nearby Support Centre for Roma and Minority Groups (SCRMG), a municipal authority providing primary healthcare, (un)employment and social services support, remedial teaching and psychological assessments.

The camp consists of two connected parts, with a different tribe residing in each one. Living conditions and hygiene are generally poor, though living standards in the eastern, bigger part of the camp are worse. Out of approximately 140 houses, 20 are brick-built and 120 consist of improvised constructions. Households are connected to a water supply network, yet there is no electricity or waste collection system available. Nearly 80% of houses depend on a generator for electricity and have absorbent cesspools as toilets in the absence of a sewage system. Food is prepared on gas cookers and heating is provided by woodstoves.

Health status of Roma minors is considered 'good' in the majority of cases, yet considered 'moderate' (50%) or 'poor' to 'very poor' (22%) for the majority of adults. 8.3% of Roma adults and 0.3% of Roma minors are known to suffer from a chronic disease. The most common diseases among Roma are: hypertension, hypercholesterolaemia, asthma, COPD, mental illness and cancer. Medication is often received through friends, rather than via a doctor. Smoking occurs in 83% of adults and 8.7% in minors, with an average age to start smoking of 13 years. Roma rely on the SCRMG especially for primary healthcare, as the nearest health centre is located 35km away.

The SCRMG pursues the integration of the Roma in the local society by taking a holistic approach to the well-being and empowerment of the population. Due to a lack of continuous support of medical staff, cases are frequently referred to nearby hospitals. Cooperation with various local/national NGOs is sought after to provide population-based healthcare services. A mediator serves as the main contact person between the SCRMG and the Roma population.

Reference

1. Census and reports from the Support Centre for Roma and Minority Groups. 2016.

B. <u>Context information based on research</u>

Information obtained during field work is provided below, supported by participants' quotes.

Centre function

<u>First place of contact between health facilities, other organizations and Roma population</u>

- *We initially refer them to our doctor here to get the first examination (…) And then, if he also thinks that something further should be done, they are referred to hospitals and so on (HP3)*
    -
- *We may have referrals by other services of the municipality, to help a family. For example, to find food or, besides... like school, to find breakfast for school, to find (school) bags, clothes, namely first aid items for basic necessities, to register maybe in social grocery store also. (KI4)*
    -
- *I: Ok. You usually visit the healthcare centre [SCRMG] or the hospital?*
CM3: Mostly the girls [the healthcare facility]. Then they tell us "for this condition you have to also visit the hospital".

<u>Provision of health education and behavioural advice.</u>

- *In case I see that a patient does not follow the medication correctly, to the extend I can, to give him/her a psycho-education regarding proper adherence to medication. (KI1)*
    -
- *Systematically, health educations being operated, by the specialties of health. Namely nurses, doctor, psychologist and so on. (KI3)*
    -
- *People, are smokers. We try to explain that it's not good, that is the reason that we have this disorder and so on (HP1)*

<u>(Free) medication for people (with lung disease) without insurance</u>

- *As social worker, the only thing I could do is to provide them (people with lung problems) with, maybe medical care, if they do not have any other type of insurance (…) I refer them to facilities in order to have their medicines, for free, regarding the conditions they have. (KI4)*

- *Medicines that we may now have in the facility may have come from people who may have not wanted them and have brought theme to us so that we can give them to people who do not have the ability to take them, neither through social insurance not through prescriptions. (HP3)*

Vaccinations for children

- *Administration of health care service and make vaccinations here, with the cooperation of our nurses. Systematically. (KI3)*

- *about children and we try to vaccinate them in every way we can (...) we perform vaccinations at least twice a month at approximately 25 children each time (HP2)*

Striking a balance between integration and health service delivery.

- *The goal of our programme is to integrate the Roma within the society (...). (If we brought more medical specialists to the health centre), what motivation would the Roma have to go to the hospital, where all people go? (...) I don't know how would this help them get to the outer world. (KI1)*

- *It is important, the population itself, to train, to be able to seek services itself [population] and not to go [close] to their door [approach] and to take for granted that they must be helped naturally [..] for them only. (KI3)*

- *We try in every case, apart from the health issues but also for the economic issues, to make them understand that they can by themselves to visit a service, ask for help, complete any task they have. To incorporate with the rest of the population (HP2)*

- *They also want this (i.e. the centre) very much, but of course we are trying to integrate them in the society, because this is the goal of the centre: to be able to integrate the people in the society, so that they are able to do everything on their own (HP3)*

Prevalence of disease

Circulatory, metabolic and mental health disorders noted as most frequent conditions.

- *The first thing, that comes to mind, as main [..] basic issue they seek to solve, is their psychiatric problems. (KI3)*

- *[...] we see many mothers who may have depressive symptoms. (KI1)*

- *I: have you happened to hear which the most common health problems are here?*
- *CM9: Cholesterol, stomach... I have both cholesterol and stomach... Blood pressure...*

Lung diseases were less prevalent. Underdiagnosis mentioned as a possibility.

- *We may see problems of diabetes, blood pressure, heart problems, respiratory problems not so much. (KI1)*

- *I think that very few people have been diagnosed (with a CLD). I mean 2 or 3 persons. And, in general, (…) the percentage of people that suffer from respiratory diseases is not high… … there is the possibility of not knowing the exact number (of respiratory patients) because they do not make appointments or undergo medical examinations, especially regarding respiratory issues (HP2).*

- *We have two respiratory disease cases, we have 2-3 allergy cases that cause respiratory problems and nothing else. Or the rest may remain undiagnosed because they have never gone (to the doctor) (HP3)*

- *I: Anything about the lungs? Have you happened to hear?*
- *CM8: No (the other participants agree as well).*
- *I: So, it is not so often?*
- *KI2: There is one case (…) my mother who has chronic lung problem.*
- *CM10: The old people have...*

Population characteristics

Lack of cooperation and strong feeling of own culture.

- *It is difficult, because it is a population with a very strong culture, which holds them back a little. (KI3)*

- *They prefer the camp (over moving into a house with their family when they can afford it). Do they feel like a group with their neighbours? Yes, this is what they feel. (KI2)*

- *…about 30 or 40 percent, I think that follow the advice of the nurse. The other is negative. I don't know why, it's a personal thing and they don't want to have a collaboration with the staff (HP1)*

- *It is that they don't co-operate. Activities will also take place now but they will be of a different level.... E.g. in the past there would be many lectures, we would perform many lectures. In the first time, 30-35 people may gather up to attend the lecture... in the second time, all of a sudden, the number would drop a lot (HP3)*

Arranged marriage at young age, impact on sexual life and education.

- *Many times, marriages are being performed without the consents of these women. So, we understand what consequences it may have when the sexual life of a girl begins at the age of 12 or 13 or 14 years old, aiming at childbearing, not love. (…) These women are married from an early age, they became mothers in a very young age, when neither their soul nor their body is mature enough to be mothers, they take responsibilities that women over 20 years old can't take. (KI1)*

- *An important thing that Roma parents have is that a girl cannot stay without marriage. For example, a girl cannot go out with her boyfriend and have sexual relationships if they don't get married. (KI2)*

- *They don't allow to get married with non-Roma people. That's why girls especially stop school very early. (KI4)*

<u>Involvement of grandparents in children upbringing.</u>

- *For the children, the grandfathers and grandmothers are mostly taking care of them compared to the parents. They are more worried. (HP2)*

- *At least (we need) to freshen the house... to make the microbes leave. Because I, and not only me, most of the grandmothers, they have their grandchildren inside the houses. (CM14)*

<u>Men work, in contrast to women.</u>

- *Most men, usually in the morning, leave to go to work [..] Women who stay back (in camp) and have the responsibility of the children also (…) Yes [there is a certain norm in the camp]. I am the man, the leader of the family, the woman stays at home with children, to take care of them. (KI4)*

- *…in the one part of the camp the women do not work (HP2)*

- *I: Does your husband work? Do you also work? Do you help him in his job?*
- *CM3: My husband and my son [work].*

<u>Increasing level of education in kids.</u>

- *Today we have an increased number of (Roma) students at school. (KI1)*

- *Due to the fact that parents understand that they cannot live only in the camp, they want to go out for sales so they can understand that without reading and writing it is quite hard to live a life. It started step-by-step that they realized that they need to send their children (i.e. to school). (KI2)*

<u>Religious (Christian orthodox) population.</u>

- *They may go to Santa Marina [a protector saint] on foot to bow down. This is also within their culture. (KI1)*

- *CM14: God may do whatever He wants... We are all close to God and may He do whatever He wants.*

- *CM9: All saints help... whatever comes to your mind... and all churches help...*
- *CM8: I have thought of Saint Ephraim and he has helped me... I mean, if someone is sick, you pray "Please make him well" and when you go to a church, you also feel better…*

Factors complicating life and potential health-influencing factors

Poor living conditions, lack of electricity and conflicts with authorities.

- *They do not have electricity systematically, or not at all, in order to have a refrigerator. (KI3)*

- *We are asking from the municipality (i.e. to fix the infrastructure) but nothing is happening. (KI2)*

- *Look, that place does not belong to the Roma. It is a place that hosts them but it does not belong to them. The idea is that "If we provide them with the infrastructure, they will stay there forever." (KI1)*

- *The antibiotics I mentioned earlier, I leave them at the mini market to store them (...) (the authorities) have thrown us like that here... The kids who also have to study (...) No electricity no nothing... (CM9)*

- *It would be very convenient for us, who are here for so many years, if they (i.e. the authorities) could fix the road, to build any sewerage disposals, so that we don't have these dirty waters, the mice running in the house (...) Also, these nylons most people have (i.e. for building their houses), we wouldn't have them... we would have built them with tins, or somewhat better... Because, they (i.e. the authorities) keep saying "We will take you out (i.e. destroy the camp)"... And people are like "I have saved 2000-3000 euros, I can't lose them (i.e. in fixing a house that will be destroyed). (CM14)*

Appendix 3. Explanation of the combined theoretical framework used for this study, including the reasoning behind development and use.

The theoretical framework we used in this study was composed from the Health Belief Model (HBM) [36], the Theory of Explanatory Models of Illness (EM) [37], and the Theory of Planned Behaviour (TPB) [38].

The HBM by Hochbaum intends to explain and predict health behaviour by focusing on beliefs of individuals. The model consists of several key concepts: the individuals' sociodemographic characteristics, the individuals' perceptions of susceptibility to disease, the perceived illness severity and the perceived benefits and barriers of performing certain behaviour. Rosenstock added the aspect of self-efficacy to the model; the perceived capability of performing the behaviour. The HBM implies that these factors, combined with certain internal and external cues to action (e.g. 'pain' or 'the illness of a friend') lead to certain health behaviour.

To help explain relations between the different factors, and explore individual's relation with the sociocultural context we added components of the TPB to this framework. The first is 'normative beliefs', meaning an individual's perception of social normative pressures. The second is 'subjective norm' which is an individual's perception about the judgement of significant others (parents, friends, teachers) towards certain behaviour. Also, the TPB includes the component of 'intention', which precedes performing the behaviour.

Both the HBM and the TPB have some limitations and can be complementary. Specifically, the limitation of both psychological models is that they focus on the decision-making individual and do not include the relational part of decision-making, nor the environmental and social-cultural shaping of health behaviour. For this reason, and also to reflect the multidisciplinary approach of the study, elements of the EM, originating from medical anthropology, were added to our framework. Specifically, Kleinmans' EM provides a useful addition to this research framework, as it addresses individuals' emotions. It focusses on the beliefs one holds about one's symptoms (illness), the personal and social meaning one attaches to these symptoms, one's expectations about what will happen to him/her, what the care providers will do, and one's own therapeutic goals. This theory therefore helps to elucidate how perspectives can differ across cultures and backgrounds, e.g. between patients and doctors.

<u>Reflection on the framework</u>

As we consolidated three validated frameworks, the overall resulting framework had not been validated before we used it in our study. We experienced the framework as effective and comprehensive. However, a more pragmatic framework may be more user-friendly.

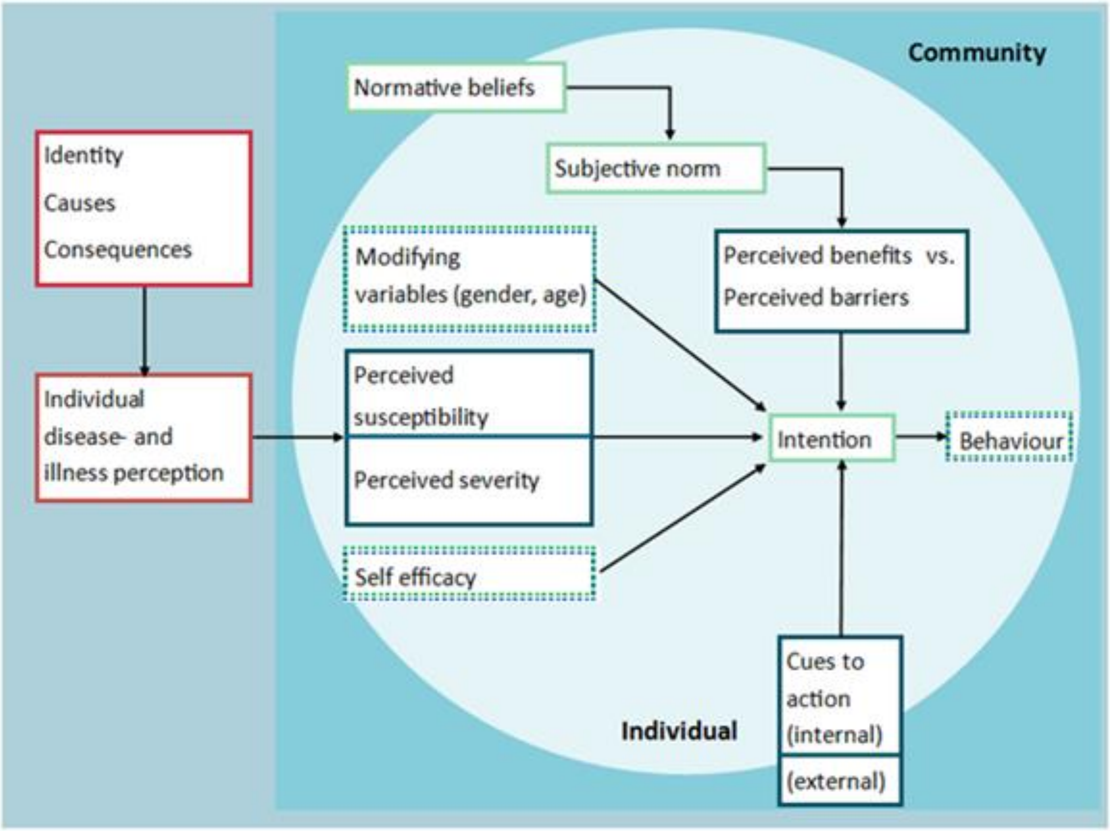

Figure 1. The combined theoretical framework as applied in the 'SETTING' tool [33], using a combination of the Health Beliefs Model [36], the Explanatory Model of Illness [37], and the Theory of Planned Behavior [38].

Appendix 4. Topic guides used for focus groups with community members and topic list for interviews with healthcare professionals and key informants.

TOPIC LIST FOCUS GROUP: Community members

Duration Interview: 60 min

A. Introduction, explanation, consent

B. Demographic data:

- Sex, age, education, education, profession (type or work, daily routine, how long does person live in community)
- Personal & cultural background (family situation- and size, composition, religion)
- External health related conditions (distance to nearst healthcare facility)

C. Introduce vignette + questions:

Story: Maria is a mother living in this village. She is 40 years old and lives with her husband and her children. While her husband works, she takes care of the children. Since three years, she notices that she becomes breathless more easily than others of her age. She especially feels breathless when she is walking up a hill. Also, she often feels tired. She has a constant cough that produces sputum, especially in the mornings. The breathlessness, fatigue and cough with sputum increase over time. Occasionally there are periods, in which breathing becomes extremely difficult. Usually such a period starts with a cold. Once she has tried antibiotics in such a severe period, but that did not seem to help. These periods usually last for a week, and then the situation improves until she feels almost the same again as before the period.

1. **Could a person like Maria be living in your community? Why is it (not) realistic?**

<identity>

2. **Please assume Maria would be living in this community. Now, as you know the local situation here best:**

What, if anything, is going on with Maria?
How would you call it?
→ **Probe**: Is she ill? If so, which disease would this be? Is there a (local) name?

Avoid a focus on TB, is necessary add that she has been tested negatively.

<causes> 3. **What do you think is the cause of these symptoms?**
(If participant named it already with 'disease' or any local term, this term can now be copied)
→ **Probe:** it's the course of life, a health problem, mental problem, external influences (e.g. the weather), working/living conditions, evil spirits, etc.
If not mentioned, could tobacco/indoor air pollution play a role?

<intention> <behaviour> <benefits> 3. **Do you think she should do something about it? Why? What should she do?**

→ **Probe:** change working/living situation, seek help

<self-efficacy> <behaviour> 4. **Who could provide help? What should he/she do about it?**
→**Probe**: family, doctor, traditional healer, community leader, church. Why this person? What would he/she do? What could a healthcare professional do? Cure or only treat? Would it be difficult to visit one?)

<own experience 5. **Have you ever noticed that you have similar symptoms as Maria? Please describe.**
'similar symptoms' = breathlessness, fatigue and cough with sputum increasing over time
(or when clearly named during the interview: replace 'condition' with a local term)

<consequence 6. **What impact does it have on your life? /do you think it has on Maria's life?**
→**Probe**: does it refrain you/her from certain work/home activities?
Does it make you/her upset/angry/scared?
Does it influence your/her social life?
Your/Her financial situation?

<severity> 8. **Do you think this is severe?**
→**Probe**: what will happen if it is not taken care off/treated (careful when to you this word)

<self-efficacy>

7. **Do you think that it is possible for you to improve your/ for her to improve her condition? Why (not)? If yes, how?**

   Do you also think it would be possible to actually prevent the condition?

<benefits/cue to action >

10. What would be reasons to do so?

→**Probe**: feel better/be able to take care of (grand)children again/etc.

<barriers >

11. What would make it difficult to do so?

→**Probe**: finance, distance to facility, stigma's

<norms>

12. What do you think others of this community think about these symptoms?
→**Probe**: stigma's? (Blame you/she for 'own fault' because of smoking? Are they afraid it's contagious? Are they supportive?) should you/she seek help?

<perceived susceptibility >

13. Who do you think will get this condition? Why do you think it does (not) affect you? How likely is it that you will get this condition?
→**Probe**: Risk factors, protecting factors.

<identity>

14. Have you heard of COPD or Asthma?
If no: → **Probe:** and chronic bronchitis? Emphysema?
If yes: → **Probe:** seen in clinic? Causes? Consequences? Big problem in community? Difficult to help? Etc.

15. See which questions should be repeated but then about COPD/asthma.

C. Field notes: (Interviewers initials, description of focus group members + characteristics, etc.)

TOPIC LIST INTERVIEW: Healthcare professionals and key informants

Duration Interview: 45-60 min

A. Introduction, explanation, consent

B. Demographic data:

- Sex, age, education, profession (type or work, total years of work experience, years of work experience in the community, type of healthcare facility), religion

C. Introduce vignette + questions:

Story: Maria is a mother living in this village. She is 40 years old and lives with her husband and her children. While her husband works, she takes care of the children. Since three years, she notices that she becomes breathless more easily than others of her age. She especially feels breathless when she is walking up a hill. Also, she often feels tired. She has a constant cough that produces sputum, especially in the mornings. The breathlessness, fatigue and cough with sputum increase over time. Occasionally there are periods, in which breathing becomes extremely difficult. Usually such a period starts with a cold. Once she has tried antibiotics in such a severe period, but that did not seem to help. These periods usually last for a week, and then the situation improves until she feels almost the same again as before the period.

4. **Could a person like Maria be living in this community? Why is it (not) realistic?**

<identity> 5. **Please assume Maria would be living in this community. Now, as you know the local situation here best, what would you say is going on with Maria?**
 -How would you call it?
 → **Probe**: Is she ill? Which disease would this be? (Local) name for phenomenon?

Avoid a focus on TB, is necessary add that she has been tested negatively.

<causes>

6. **What do you think is the cause of these symptoms?**

(If it has been named by participant already with ‘disease’ or any local term, this term can now be used)

→ **Probe:** it’s the course of life, a health problem, mental problem, external influences (e.g. the weather), working/living conditions, evil spirits, etc.

If not mentioned, could tobacco/indoor air pollution play a role?

<causes> **What do most people in the community think causes these symptoms?**

<intention> <behaviour> <benefits>

7. **Do you think she should do something about it? If so, what should she do?**

→ **Probe:** change working/living situation, seek help

<self-efficacy> <behaviour>

8. **Would you be able to provide help? What would you do?**

→**Probe**: would you be able to treat it or cure it? Upon what does the success of treatment depend?

<own practice>

9. **Do you ever have people visiting you for the same condition as Maria has?** ‘same condition’= breathlessness, fatigue and cough with sputum increasing over time

(or when clearly named during the interview: replace ‘condition’ with a local term)

<severity> <consequence>

10. **Do you think it is severe?**

→**Probe**: what will happen if it is not taken care off/treated (careful when to use this word)

What problems may it bring to Maria, and to the family? In terms of health, money, or socially

<self-efficacy> <behaviour>

<self-efficacy> <behaviour>

11. **Do you think that it is possible for you to improve her condition?**

Why (not)? What would you do?

<own practice>

-Diagnose

-Treatment

- Prevention

→**Probe**: why do you do it this way? Is this also the way you ideally would do it? Why (not)? (lack of knowledge, resources, guidelines?, etc.)?

<benefits>
<cues to action>

9. What would be reasons for you to help her?

→**Probe**: job position/community respect/salary, etc.

<barriers>

10 . Which problems have you experienced in helping people like Maria?

→**Probe**: lack of access to resources, lack of compliance, etc. Is it difficult for people like Maria to visit a healthcare worker? (access to healthcare, money, communication, dislike treatment?)

<norms>

11. What do your colleagues about helping people with these diseases?

→**Probe**: stigma's? (Blame you/she for 'own fault' because of smoking? Are they afraid it's contagious? Are they supportive?)

<

<perceived sceptibility>

12. Who do you think will get this condition? What these people and not others?

→**Probe**: E.g. children/elderly, men/women/ etc. Risk or protective behaviour

<perceived sceptibility>

13. How likely is it that people in the community will develop this condition? (or, when clearly named during the interview apply local term).

→**Probe**: How likely, why (not)?

<identity>

14. Have you heard of COPD or Asthma?

If no: → **Probe:** and chronic bronchitis? Emphysema?

If yes: → **Probe:** seen in clinic? Causes? Consequences? Big problem in community? Dificult to help? Etc.

15. See which questions should be repeated but then about COPD/asthma.

C. Field notes: (Interviewers initials, description of focus group members + characteristics, etc.)

Appendix 5. Supplementary quote box providing additional quotes supporting the identified themes.

**Awareness and CRD-related beliefs**
▪ *They (i.e. Roma people) will say "I have a problem with my lungs". They don't know names (...) they stay to more simple names like "A problem with my lungs", "I cough". (HP3)*<br><br>▪ *Our family, everybody (has allergies). All winter we are like "achoo" (makes the sound of sneezing), rubbing our eyes, taking drops in the eyes, putting spray in the nose. (CM14)*<br><br>▪ *Yes, he (i.e. her son) got tired. Like augmented, I took him (i.e. to the doctor) and they told me, that the child has asthma. (CM1)*<br><br>▪ *Interviewer: And have you happened to hear this second name: COPD?*<br>*CM8,9,10,11: no, no...*<br><br>▪ *Is it hereditary (i.e. the vignette situation)? Maybe someone had it [relative] and he inherited it (...) Maybe some allergy could cause this, the flowers, dust... (CM1)*<br><br>▪ *I believe that it comes with age... Because everything appears when you get old... And from smoking! There are some people that when they have it (i.e. the lung problem) they quit smoking. (CM8)*<br><br>▪ *I hear that smokers get sick and their liver (probably means lungs) gets black. (CM3)*
**Symptom severity and disease susceptibility**
▪ *Because everything comes from the lung... All people should take care... maybe your breath will stop... this is what I know... (CM8)*<br><br>▪ *Interviewer: Generally, if someone coughed a lot and frequently, would it be considered normal?*<br>*CM3: They would go to the doctor.*<br><br>▪ *It affects her (i.e. the woman in the vignette), yes… If she has this asthma, it will continue for long time. (CM1)*<br><br>▪ *They (i.e. Roma) believe it (i.e. breathing problems due to woodstove smoke) is normal. (KI1)*<br><br>▪ *If you want to live for many years, you have to quit smoking! (CM8)*
**Smoking behaviour and HAP exposure**
▪ *These people also consume much tobacco, they smoke very, very much. (KI1)*

- *CM3: I believe that they smoke from very young age. They start early.*
  Interviewer: At what age approximately they start smoking?
  CM3: 13, 14, 15.
  Interviewer: Do you share the same opinion? [referring to CM4]
  CM4: Yes. Because in our area, the children start smoking early.

- *Interviewer: Do most people smoke inside or outside the house?*
  CM14: Inside. Our babies also smoke inside (i.e. refers to passive smoking).

- *Interviewer: So, do most of the people have the woodstoves?*
  All: yes...
  Interviewer: And do you use it for many hours to cook?
  CM10: All day long... if you have woods to put in...
  Interviewer: So, you keep it lighted up, to have warmth but also to cook, right?
  CM8: Yes, because most of the shacks are built with tins and they can't get warm... it is very difficult...
  CM10: And we light it up all day long and during the night.

- *Interviewer: Do you try to ventilate the house? (...)*
  CM3: We don't take much care of it. I close the windows and the door sometimes (...) We all sit close to the woodstove. And I don't open something to ventilate the area. Only when the weather is good....

Healthcare seeking behaviour

- *If (breathlessness) is something that persists and they have it systematically, yes. However, if they feel it rarely or only when they do certain activities, they will forget about it (...) when their health worsens, when they need hospitalization or to visit the emergency department, only then they realize it and search more (for help). (HP2)*

- *Interviewer: But when will they go to the doctor or the hospital?*
  CM14: When they fall in bed and can't get up.

- *Interviewer: ...you had some breathing problems... what did you do? did you go to the doctor?*
  CM10: No.
  Interviewer: Did you ask anyone in the family? Or friends?
  CM10: No, there is just a doctor who comes at the facility, but only the older people go there for their health... us the younger ones don't go... we just sit wherever it's cooler.
  Interviewer: I see... But do they (i.e. the breathing problems) pass?
  CM10: Yes, when you are at a cooler place you take some oxygen.

- *I don't leave my child without going to the doctor (...) whatever the doctor says, I do everything (...) If I have 100 euros, I will prefer to give them for my kid (...) For myself, I will go to the hospital. But (...) even if I don't go, I have no problem. (CM15)*

- *I have interpreted it this way "Even if I hurt, I am not going (i.e. to the hospital)" (...) it's not that the person is not interested about health but rather that most of them are afraid of what they will hear from the doctor. (KI1)*

▪ *(A Roma man may be like) "If I go to the hospital, they may find that I have a severe condition. I will not go, I will stay at home and whatever happens" (...) Or they may fear the hospital and whenever they go they may feel bad. (CM15)*<br><br>▪ *There is no interest (...) Especially for health-related issues (...) In general, I consider that they are not taking care of their health except if it is something that needs immediate care or hospitalization (HP2)*<br><br>▪ *(...) health is not important for them. It is not a priority. Important for them is to work, make some money and have food today. (HP2 about CMs)*<br><br>▪ *When we have a nursing activity, along with a lecture, e.g. when we perform spirometry (...),_ and we also have the doctor who will see their examination and will talk to them and refer them to the hospital, this more interesting for CMs… and it is out of discussion that if the benefit was financial, it would be much better (...) This is more motivational, it makes everything more interesting. (HP3 about CMs)*
**Perceived barriers and facilitators to care**
▪ *…they stay in the shacks. Cooking or heating with a particular manner. Maybe these also affect their respiratory (health)… During winter (...) The truth is that they also do not have the means to do it in the place that they live (i.e. change cooking/heating conditions). I mean the economic condition (HP2)*<br><br>▪ *(...) they feel uncomfortable and disadvantaged because they are illiterate, to visit a (health) service and undergo some tasks. Their level of education, I think, draws them back because they feel uncomfortable and disadvantaged. (HP2)*<br><br>▪ *(...) when someone gets something, an escort of even 10 people may follow him/her (i.e. to the healthcare facility) (...) they have an "audience", they never go alone for something they consider very important. (HP3)*<br><br>▪ *If we see someone that is sick and cannot afford to go to a hospital, and he cannot go, we ask him if he wants to help him to go to the hospital and offer money from our own. (CM4)*<br><br>▪ *…it is easier for them to come (at the Centre) because it is close. But when we sent them elsewhere, they might say they don't have gas, or the money for the tickets or vehicle to go and many times we arrange appointments that they do not attend. (HP2)*<br><br>▪ *We have created a relationship of trust and we are somehow a reference center for them. (KI1)*
